# Exploring socioeconomic inequality in educational management information system: An ethnographic study of China rural area students

**DOI:** 10.3389/fpsyg.2022.957831

**Published:** 2022-09-09

**Authors:** Qing Ye

**Affiliations:** School of Educational Sciences, Harbin Normal University, Harbin, China

**Keywords:** SEI, conflict theory, EMIS, ethnographic research, exploration

## Abstract

There is currently enough systematic literature presents about socioeconomic inequalities across different disciplines. However, this study relates socioeconomic inequality (SEI) to rural students educational management information systems (EMIS) in different schools in China. The dynamic force of information technology could not be constrained in the modern techno-based world. Similarly, the study was qualitative and ethnographic. Data were collected through an interview guide and analyzed with thematic scientific analysis. Ten male and ten female students were interviewed based on data saturation point. The purposive sampling technique was used for the rural school and students’ selection. This study summarizes the findings and brings together in-depth emic and etic findings based on new Marxist conflict theory, exploitation, and domination power lens. The study found that SEI creates disparities among EMIS. Household income inequality has influenced on educational achievements of rural areas’ students. Gender-based SEI was not present among students. Family wealth and SES-based exploitation are present regarding EMIS among male and female students. Household wealth is significant for the EMIS. The study put forward a recommendation to the policymakers that exploitation could be overcome among students if the government provides equal opportunities for access to the EMIS.

## Introduction

**FIGURE 1:**
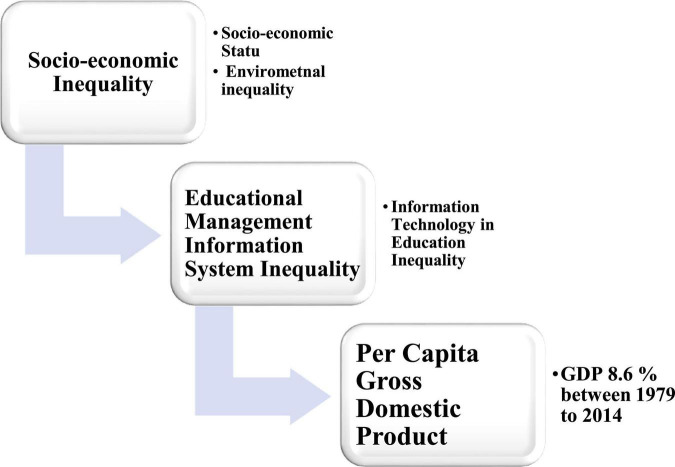
Conceptual model of interlinking disparities process.

The focus of my study is to explore the socioeconomic inequality (SEI) and educational management information system (EMIS) among rural background students in China. Nowadays, China’s fast economic expansion has come at the cost of increasing socioeconomic disparity and a low level of information technology in education. Similarly, the SEI is also a relationship with the capita gross domestic product (GDP), and it increased to 8.6% between 1979 and 2014. The improvement in ordinary people’s living conditions remains modest, and the wealth gap between the affluent and the poor is expanding (Cheuk et al., 2021). Only one percent of Chinese families had more than one-third of total household wealth and education in 2014, while the poorest one-fourth owned less than 2% (Xie and Jin, 2015). Meanwhile, inhabitants in some areas of China have been exposed to substantially increased risks of severe weather and environmental pollution and access to education as a consequence of aggressive economic growth (Liu et al., 2010; Cho et al., 2022; Ye et al., 2022), threatening their quality of life, education, and health (Han et al., 2021). Furthermore, human is expected to rise to 1,563/million persons per year by 2060 in China. Meanwhile, in 2060, the loss of GDP due to an increase in health spending, access to education and information technology, and labor productivity are expected to rise by around 2.1% Organization for Economic Co-operation and Development (OECD) (OCDE et al., 2016). Figure 1 discusses the conceptual understanding of the interlinking disparities process in the picture form.

**FIGURE 2:**
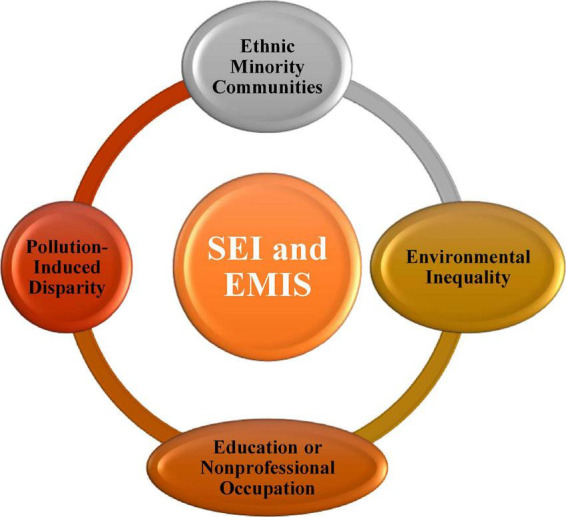
Cyclic process of socioeconomic inequalities regarding the EMIS.

The idea constitutes a new domain with largely unstudied potential in the systematic literature because there is a SEI problem in the sector of education of EMIS. The interconnection of SEI and the EMIS academic field matures with qualitative methods and techniques through the ethnographic lens. SEI has been the object of various studies in the last two decades, and it has a direct relationship with information technology as well as education. In light of this, in Chinese cities with the fastest expanding economies, such as Beijing and Shanghai in China, pollution-induced disparity and accompanying environmental inequality (EI) between the affluent and the poor may be seen (Xie and Jin, 2015). Nevertheless, SEI and EMIS are still missing in the perspective of students, and it is characterized as disadvantaged academic domain, for instance, ethnic minority communities or low-SES and SEI rural groups bearing a disproportionate distribution in the domain of education and their access to the information management system (IMS). A considerable body of research has been conducted in exploring the people with low socioeconomic status (SES), low income, and low education or non-professional occupation are more likely to experience, but the IMS is a higher level of influence on the education, which could decrease environmental catastrophes, such as air pollution, flood, drought, and extreme heat (Li et al., 2018; Park et al., 2018; Ur Rahman et al., 2021; Zhuo et al., 2021). Hajat et al. (2015) concluded that the above scientific pieces of literature have concentrated on the effects of EI on these three primary SES indicators without taking into consideration possible influencing variables such as family income, education, and MIS. Vassilakopoulou and Hustad (2021) examine the most recent decade’s worth of information system (IS) research on the digital divide in the high technical infrastructures and economic conditions. It was found that models of digital disparities were present, and the SEI factor impacts the digital divide among different societies. This particular ethnographic qualitative research paper found the gap in the previous literature and then drew the cyclic process of socioeconomic inequalities regarding the EMIS for rural background Chinese students, which is depicted in Figure 2.

As a consequence, these studies may have failed to explain SEI and the consequent differences in SES effectively. Several social epidemiological factors are missed in the domain of SEI, such as wealth and income, education, and IMS, which are a stronger indication of wellbeing inequality in society, especially for a new generation of students labeling. Over the last two decades, several academic authors have claimed that environmental consequences disparities and family wealth, rather than household income, are more robust indicators of SES in China (Pastor-Satorras et al., 2015; Chu et al., 2020). The family’s wealth may represent one’s capacity to acquire an apartment in one’s chosen location, which could influence the household members’ exposure to education and its requirements in the technological world. To acquire a better knowledge of SES and SEI-based in China, it is necessary to look at whether different SEI has an impact on the EMIS and its exposure in general among students (Zheng and Yin, 2022).

**FIGURE 3:**
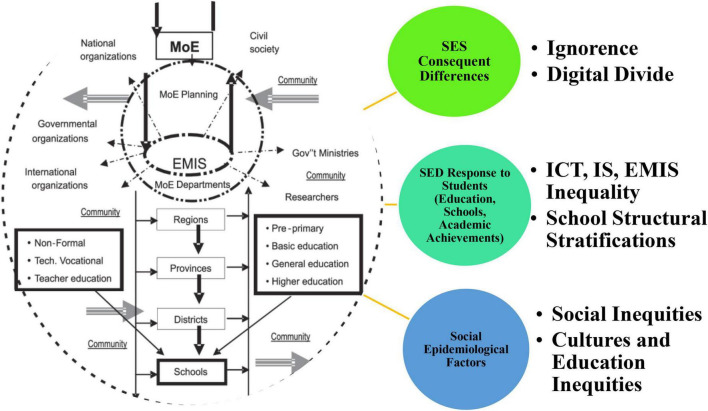
Conceptual perspectives of advanced literature and in-depth themes representation.

This is a problem posed in terms of SEI and EMIS among rural students of the middle level (Figure 3). The importance of SEI research done at the rural-level students captures the critical spatial factors of EMIS, which is still ignored in a high gross domestic product (GDP) growth country China. For instance, Wang et al. (2021) pointed out that Information and communication technology (ICT) had a significant influence on the economy and society in recent decades. More precisely, although ICT is critical for promoting socioeconomic development (SED), and its detrimental impact on SED in neighboring regions (education, schools, and academic achievements), meaning that China’s provinces have a digital divide that might lead to high socioeconomic growth and the inequality remains constant. The research concluded that some practical policy proposals for the growth of ICT in the future are important to finish inequality among rural communities based on EMIS, reduce the negative impacts of the digital divide, and maximize the advantages of ICT-based SED is possible. Furthermore, Zehavi et al. (2005) found many social inequities exposed by several academic studies. However, most rare, the interaction and entanglement of digital technology, structural stratifications, and the established propensity of “othering” in cultures of education especially using the lens of an intersectional feminist approach, are narrated minor, which is a dire need in the China rural background. We propose that IS research move beyond simplistic notions of digital divisions to examine digital technology as implicated in complex and intersectional power systems and improve our sensitivity to the positionality of individuals and groups within social orders as part of a future research agenda (Zheng et al., 2022). There are other implications for practice and policy, such as going beyond the single-axis analysis of digital exclusion and students’ education related to IS s were an excellent lens to study in the future. In light of this, Stewart (2021) argued that academic institutions, academics, administrators, educators, and students have thoroughly appreciated the emergency remote teaching (ERT) strategy. The global world started academic communities throughout switch to ERT. This literature overview combines the four significant themes that emerged from a thematic analysis of the findings. Such as ERT experiences, digital divide, and massive educational/socioeconomic disparities, routinely encountered ERT difficulties, issues, and challenges, and frequently made ERT changes. The study recommends to future researchers that technology is the best tool to teach students without socioeconomic inequalities (Ma et al., 2022). This problem is a long-standing challenge for the Chinese rural students and communities, especially in the sector of education, which could be counted with these particular remedies (see Figure 3).

## Theorizing social class

**FIGURE 4:**
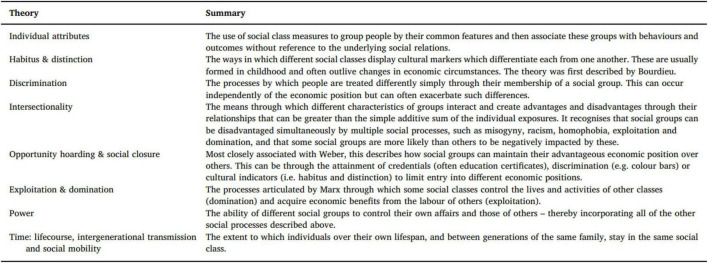
The exploitation and dominance of the people in social classes (reproduced with permission from Krieger, 2001).

The concept of social class was coined by Karl Marx, and Krieger (2001) explained the socioeconomic domain in the form of social class. Social class is a person’s economic contacts that lead to the formation of social groups. The production, distribution, and consumption of goods, services, and information, as well as the relationships between them, affect these interactions. As a result, social class is founded on a person’s position in the economy, whether as an employer, employee, self-employed person, or unemployed person (in the formal and informal sectors alike). Furthermore, the exploitation and dominance of the people are part of social class. These class nominations create some inequalities among individuals and societies, which are elaborated in Figure 4.

## Conflict theory of extension in the form of exploitation and domination

**FIGURE 5:**
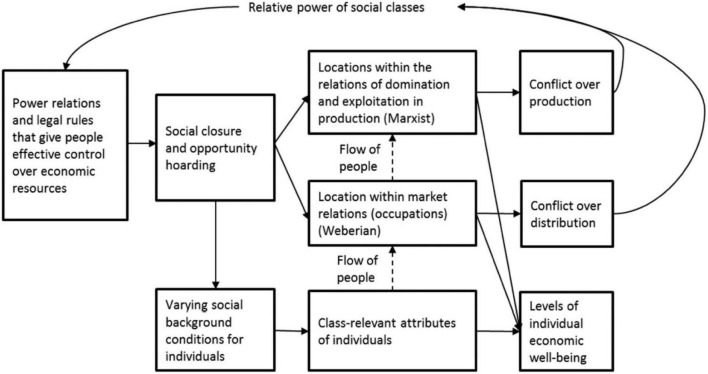
Relative power of social classes theory and the socioeconomic inequality (reproduced with permission from Wright, 2015).

Wright describes the relation between exploitation and domination as part of class theory. This conceptualization is most closely aligned with Marxism (or neo-Marxism) (Muntaner et al., 2002; Muntaner and Lynch, 2020), and it describes the processes by which some social classes control the lives and activities of others (domination), as well as the processes by which capitalists (owners of the means of production) gain economic benefits from the labor of others (exploitation) (Wright, 2015). In this perspective, the main distinction between social classes is between those who own and control the means of production and those who are paid to utilize them. Additional subcategories may be added education of the parents, and their children have full exploited in this way; the educational capabilities of non-dominate class students do not grow because they have no access to a high level of education and technology (Breen and Goldthorpe, 2001). The theory of exploitation and domination is applied to the SEI among Chinese rural students related to EMIS. Some classes have all educational opportunities in information technology, a modern school system, a high level of facilities, and wealthy living. On the other hand, some students have no access to these facilities due to low SES. Similarly, the lens of social class theory is deductively explaining the importance of EMIS for rural background students in China (Ma and Zhu, 2022). In the light of this, the Wright relative power of social classes theory is more powerful in overcoming the socioeconomic inequalities among societies (Wright, 2015; see Figure 5).

For an overview of existing techniques, the study is directed to solve the SEI and EMIS with help of social class theory and its subtheory of exploitation and domination of class. It is to be noted that the importance of SEI is not ignored in the domain of EMIS because family wealth, economic status, and its relationship with students’ education are challenging topics in Chinese academic research. This is a common problem encountered while using such a qualitative method to explore the in-depth understanding of the SEI and its interconnected challenges with China rural students at the middle school level (Chen et al., 2021). China is growing to overcome such social epidemiological challenges for the urban students, but the part of the rural students is ignored, and this in-depth, subjective study explored the challenges and solutions from the emic and ethical perspective of the students. Second, we examine the existing Chinese home wealth database and highlight current research limitations, such as the absence of high spatial resolution and economically representative family wealth data that may aid new a domain of EMIS research in China. Third, using a qualitative methodology, we explore the various methodologies (e.g., emic, and etic perspectives) for constructing appropriate SEI proxies throughout the research, which is also highlighted by the global educationist for solving such type of phenomenological challenges in the rural background students. Fourth, concerning SEI and EMIS research in China, we address the advantages and disadvantages of current new SEI proxies for assessing SES, household wealth, education access to all, and its relation to the social class theory of Karl Marx. Fifth, in relation to SEI proxy development in China, we summarize the challenges to data availability and quality, including ethical and privacy concerns, and recommend that policymakers improve the quality and availability of MEIS while removing the SEI in the provision of the IS at school level (Xiong et al., 2022). Finally, we wrap up our research and provide recommendations for future research into new SEI proxies to aid EMIS investigations in China to overcome student socioeconomic disparities.

## Research design

**FIGURE 6:**
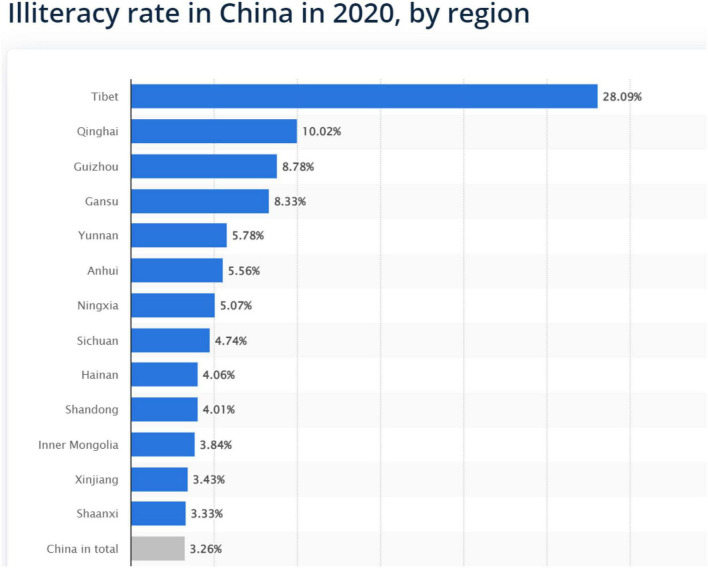
The qualitative research data for the illiteracy rate in China (reproduced with permission from Hannum et al., 2021).

The qualitative research process was ethnographic. First, we reviewed the Chinese and worldwide literature to construct SEI, SES, EMIS, IS, and IT and then systematically specified the studies emphasizing the social class, domination, and exploitation related to these specific themes. Consequently, the theoretical framework was developed to position the debate on inequalities and their relationship with rural students in China. The population and sample of the students were taken from China one province of the total. In this particular study, we selected Hainan Province rural areas school and their students (see Figure 6).

In this research, the interpretive viewpoint was applied. A subjectivist presumption, which generates reality within a social context, is the foundation of the interpretive viewpoint (Bell and Bryman, 2005). The research used a constructivist methodology. This method leads to the epistemological underpinning of the method (Davis and Sumara, 2002). Similarly, Guba (1989) distinguishes between conventional and constructivist belief systems, in which socially created realities are based on the society’s dominant belief system and are viewed and understood differently by many people. Natural rules do not regulate socially produced reality, which is a truth. When an individual’s view is based on a single fact, it is not acceptable; alternatively, a consensus of persons is acceptable under a constructivist approach that emphasizes truth. Constructivist views rely on a monistic subjectivist epistemology that postulates questions, with humans posing these questions about the social environment and then discovering the final answer in their own time (Guba and Lincoln, 1989). As a result, it is shown that dialectical repetition employs a hermeneutic technique that is considered constructivist. Similarly, analysis and criticism, reiteration, reanalysis, and recritique are pragmatic criteria for reaching logical knowledge and building strong thinking skills. The research was based on the author’s subjective interpretation of earlier ideas on the link between Chinese middle school rural students’ socioeconomic disparities, education, and EMIS. The laddering methodology, which was further explored in the data gathering procedure, was used to eliminate bias from the data. According to Bell and Bryman (2005), the interpretative viewpoint is commonly employed in qualitative research. According to Eriksson and Kovalainen (2015), it is conceptually reliant on explanation. The quality of interpretative research focuses on human sensibility and complexity rather than preset categories and variables (Eriksson and Kovalainen, 2015).

In this study, a qualitative ethnographic technique was applied. The ethnographic study is a way for researchers to get a more profound knowledge of a field by immersing themselves in it to build in-depth information and analyze the people’s culture and social environment. Its goal is to “make the unfamiliar familiar” through “making sense of public and private, overt and obscure cultural meanings” (Grech, 2017). A sample of 10 male (middle school students) and 10 female (high school students) students were chosen. The students from the rural region ranged from 11 to 14 years old, and both male and female students took part in the research. The respondents had similar features such as age, class, rural school system, and government educational system, and they were chosen purposively among school students (population). Participants were from a rural background. It should be highlighted that all male and female students were from low socioeconomic levels (SES), except one student who was from high SES in this study. During the interview, the participant described this information.

**FIGURE 7:**
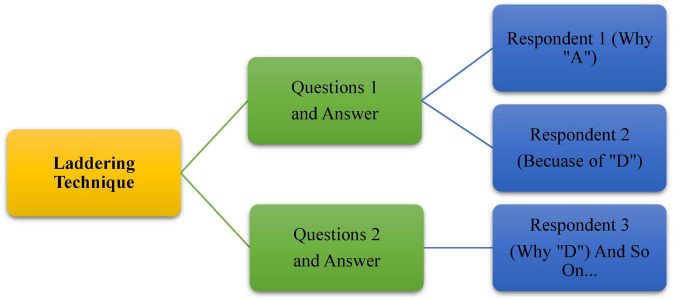
The laddering approach to explore the participants’ behavior (reproduced with permission from Veludo-de-Oliveira et al., 2006).

In-depth and unstructured interviews were used to gather information from participants. Voices, knowledge, and perspectives are prioritized in this strategy (Smith, 1999). During the interview, a laddering strategy was also applied. It is one of the psychological interview strategies that’s quite useful for field research. The laddering approach has the advantage of allowing researchers to explore the participants’ behavior. “This strategy entails asking the interviewee follow-up questions based on their prior responses to acquire a better understanding of the respondents’ perspectives” (Veludo-de-Oliveira et al., 2006; see Figure 7).

The interview lasted 30 min and included all of the participants. The interview was done in Chinese to understand the respondent’s opinions fully. It was then translated into English to report on this research. The school heads and their parents gave their informed agreement, and then the students were asked to agree to an interview.

In this particular study, the rural schools in the Hainan province of China students were interviewed, and the schools’ names are below:

“Tengqiao Middle School in Sanya Haitang District, National Middle School in Jiyang District, Meishan Middle School, Meishan Primary School, Baogang Middle School, Yacheng Middle School, and Nanbin Primary School in Yancheng District.”

**FIGURE 8:**
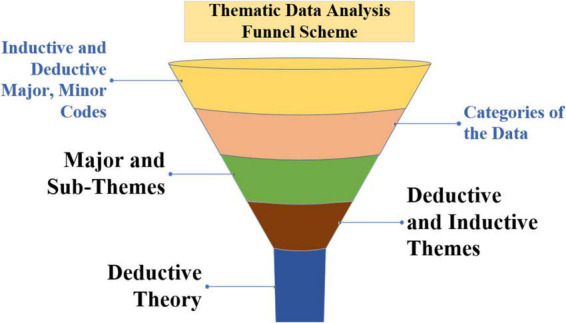
The thematic data analysis funnel system.

During this period, the participants were advised that their real identities would not be revealed in the reports and that pseudonyms would be used instead. The theme analysis approach helped assess outcomes in earlier investigations. This strategy may provide the reader with detailed, contextual, and culturally sensitive facts. The thematic analysis technique was employed for data analysis, discovering, interpreting, and reporting patterns or themes within acquired data. In this case, narratives were used to describe the outcomes, which helped to clarify them (Braun and Clarke, 2006). One of the benefits of thematic analysis is that it allows researchers to identify patterns in the respondents’ statements via a flexible, inductive, and ongoing process of connecting with narratives. As demonstrated in the thematic data analysis funnel system picture (Figure 8), all context is categorized into fluid categories of deductive and inductive themes, subthemes, and coding.

## Data analysis and findings

**FIGURE 9:**
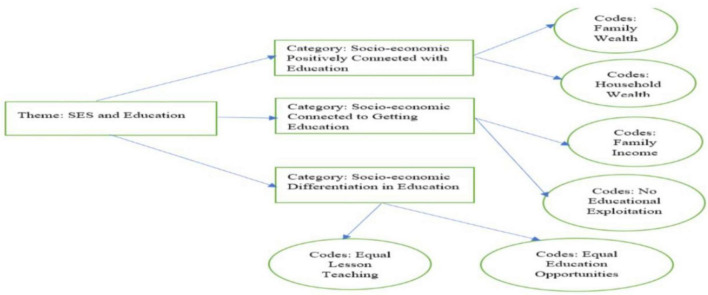
Theme: The socioeconomic status and education.

The results, which portray the genuine voices of participants and all names and identities, are classified for anonymity and confidentiality, are shown below. The theoretical link between rural students’ SEI viewpoint for EMIS and its relationship with social class, dominance, and exploitation is written in the data analysis section (Figure 9).

The participants replied that income, education, and employment are typically used to establish traditional metrics of SES. Similarly, some participants claimed that household SES had influenced their educational achievements during school. Such as, we could not use information technology because we have no exposure and other schools in the urban areas have information technology. Furthermore, some respondents described that family wealth is essential and then we will get a good education. Most schools in the urban area have access to education and information technology. The real verbatim is written below:

“Chinese version (我希望我父親是個女帽匠, 我在高級學校系統學習 Wǒ xīwàng wǒ fùqīn shìgè nǔ mào jiàng, wo zài gāojí xuéxiào xìtong xuéxí) (EMISS-2).”

“I wish my father would be a milliner, and I study in the high-level school system” (EMISS-2).

Furthermore, the participants stated that our middle school is good, and we have information technology without discrimination against SES. Family affluence may impact the individual but not the school, and the Chinese government gives almost equal education opportunities.

“I am happy with my parents’ socioeconomic status, and there is no difference in the school regarding getting an education” (EMISS-10).

Some participants suggested that the impact of household wealth on the student’s exposure regarding education is more. The participants might have been readily explained by household income, representing a family’s economic wellbeing, and positively connected with education exposure. The distribution of economic values represented substantial divergence for the students in the school. Family wealth is more unequally distributed in urban and rural China. In this regard, one student discussed that household income might be better for getting an education, and SES could create disparities among rural and urban areas students’ education. The real words of the respondent have narrated below…

“I noted that my classmate has high parents’ SES, and his teacher taught him after his school time. I wish that I should learn many languages, such as the English language and the American English Language accent, but I could not” (EMISS-20).

According to some participants, there is no strong connection between parents’ high SES and access to education. Teachers are from the same family income, and they have not created disparities among students regarding the lesson’s teaching. On the other hand, participants replied that household wealth or income becomes a more important SES indicator for educational exposure and getting an education. Given its more excellent stability and more prominent effect on living standards over time, family wealth may reflect a higher degree of education.

“I know there is no educational discrimination because of parents’ SES in middle school” (MISS-15).

**FIGURE 10:**
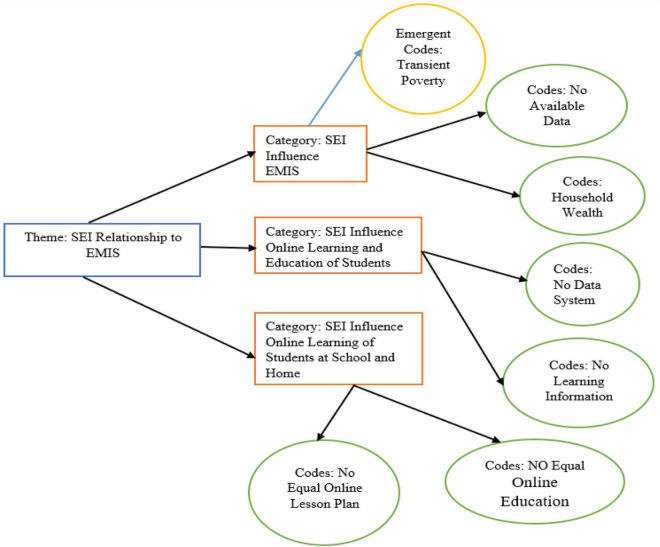
Theme: Socioeconomic inequalities and educational information management system.

Because of the issue of socioeconomic disparity in educational achievements, this concept represents a novel and mostly unexplored area of research in the academic literature. In light of this, Jiang found that SES has influenced China’s students’ educational achievements (Jiang, 2021). Pesando (2021) claimed that social consequences and family wealth for getting an education are more significant predictors. The current study found that SES has a relationship with educational achievements. SES and family prosperity may have an impact on educational achievements. Furthermore, the household and SES influenced students, educational achievements at the school level (Figure 10).

The participants revealed that income shifting does adequately reflect living standards and also does influence the educational achievements of middle school students. Some students agreed that socioeconomic inequalities exist in the students, and some students have a good understanding of EMIS compared to low-income students. The high socioeconomic students can buy computers, laptops, and mobile to get online education. Household wealth inequalities reflect structural and chronic poverty, which further stop students from learning in the online education system. Similarly, household wealth inequality is the form of transient poverty, which is less volatile and more reliable. Wealth, rather than income, is a better predictor for EMIS and information technology.

“I wish that I have a fast computer and internet connection for the learning of education” (EMISS-12).

However, the participants were from industrialized family backgrounds, and they described the socioeconomic inequalities that exist in some school systems. The city school system was different from online information-sharing system, and the city school gave all the facilities to their students whenever we were studying online. Now, we shifted to rural area schools, and there is no such reasonable EMIS for students learning.

“I believe that the city school system was sound good, and its EMIS performance was better than the rural school system. My parents shifted village home from the urban industrial areas, and now I feel a difference regarding EMIS and information system in this rural area school” (EMIS-18).

The participants agreed that some of our classmates dropped out of school because of their parents’ low household income, which is a dangerous sign for the overall personal career of the students. Income drops the family’s living standards, and their students do not remain relatively consistent with getting an education. The participants conditioned here and said that if household income is more robust, it is a good sign for the students’ once future or personal careers. The true words of one participant were quoted. “I agree that socio-economic inequality can stop some from getting an education in their career” (EMIS-9).

The relationship between EMIS and family salary is interrelated with the instructive career. Members advance cited that family salary makes a great pointer to one’s mental capacity since, without the pressure of money, a person can examine and teach very well. Within the final month, a few students’ guardians were challenged within the Hainan territory for their children’s data innovation get to and web of things. These guardians were from the moo SEI, and they might not give EMIS contraptions to their understudies. Moreover, a few members have talked about how long-term fabric amassing is superior to short-term wage since long-term fabric accumulation sustains the children’s instruction, and they can purchase EMIS contraptions for way better learning results within the school. The moo SES of understudies certainly uncovered the non-appearance of different data technology-related contraptions.

“I have no information technology-related tools for accessing the EMIS system. The school is closed, and our educational activities are not sustained due to no computer system and mobile phones for the online management information system” (EMISS-7).

**FIGURE 11:**
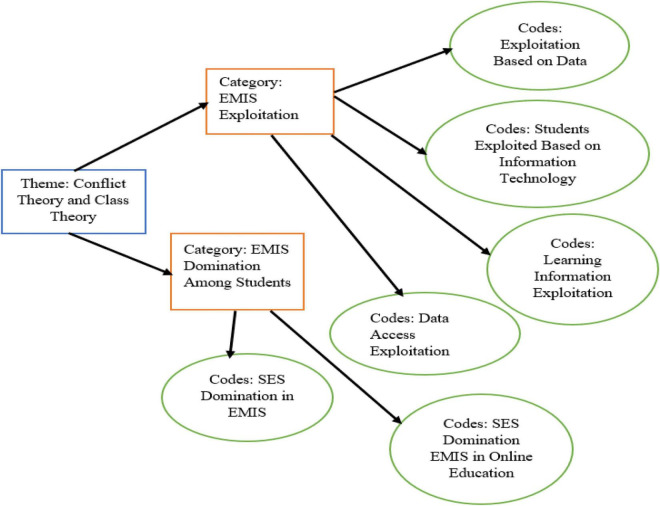
Theme: Conflict theory of extension in the form of exploitation and domination.

Wang et al. (2021) revealed that ICT significantly influenced the economy and society in recent decades. Although ICT is critical for promoting SED, the inequality of the digital divide is present. Furthermore, Zehavi et al. (2005) found that many social inequities and digital technology interactions are different in the structure of society, which influence educational culture among students. Stewart (2021) argued that academic institutions, academics, administrators, educators, and students have thoroughly appreciated the ERT strategy. Such implementation is not fruitful due to socioeconomic disparities in the educational institution. The results of the current study were in link with the previous literature. Similarly, SEI exists in the students, and high-income students have a good understanding of EMIS compared to low-income students. Likewise, high socioeconomic students have the capacity to buy computers, laptops, and mobile to get online education (Figure 11).

Participants replied that EMIS exploitation and domination are present to some extent. Similarly, some participants claimed that low socioeconomic students are exploited based on online educational learning. Similarly, we could not use information technology because we have no exposure and other people of high economic status have information technology access in their homes. Furthermore, some respondents described that EMIS is essential for getting a good education. Most students have access to information technology. The authentic verbatim is written below:

“我希望家裡有信息技術小工具，我不會在課堂學習中被剝削.”

“Wǒ xīwàng jiāli yǒu xìnxī jìshù xiǎo gōngjù, wǒ bù huì zài kètáng xuéxí zhōng bèi bōxuè” (EMISS-16).

“I wish would have information technology gadgets at my home, and I would not be exploited in my class learning” (EMISS-16).

The participants answered that we have no access to EMIS, and our education is exploited due to no access to information technology, and it is one sort of discrimination based on low SES. Family exploitation may impact the student’s education at the school level, and the Chinese government should give information technology tools to every student to save their academic life.

“我對自己的教育成績不滿意，因為去年我因為無法使用信息技術或 而沒有上課.”

“Wǒ duì zìjǐ de jiàoyù chéngjī bù mǎnyì, yīnwèi qùnián wǒ yīn wéi wúfã shǐyòng xìnxī jìshù huò EMIS ér méiyǒu shàngkè” (EMISS-6).

“I do not feel happy with my educational grades because last year, I did not attend classes due to no access to information technology or EMIS” (EMISS-6).

The study participants suggested that exploitation in the school influences students’ exposure to EMIS. Dominant household income students represent their selves in the school, and their EMIS exposure is higher than low household income students. For the students in the school, the distribution of economic values showed a significant disparity. The information investigation appears how complex the exchange between the person, his social context, and the instructive framework truly is. It too uncovers the tirelessness of the meritocratic perfect of person organization in instructor, parent, and indeed student discourses. However, at the same time, the problematization of minority and working lesson habitus and the culturalization of “educational failure” appears to drag the plug out of this argument, because it presupposes that a person’s understudy is (emphatically) decided by his or her domestic environment. From that viewpoint pupils’, parents’, and indeed teachers’ agency as it appears to play a minor part. In this talk, we begin with expanding some limitations and preferences of our paper, whereas in a moment area, the broader social implications of the discoveries are talked about the urban and rural China education system is unequal, and their family SES is unequally distributed. This reason creates dominancy among students, and low SES students have no access to the EMIS at home during online classes. The true words of the participant have narrated below…

“I noted that my classmate has a dominant level in the class, and she has access to the current EMIS in the home. I wish that I should learn about EMIS in the school” (EMISS-11).

Moreover, the participants revealed that the dominant attitude of the students is due to high SES in the class, and they already have access to the EMIS. EMIS and parents’ high SES have a strong connection to their educational achievements. Teachers are from the same family income, and sometimes they exploit students of low SES in the classroom. On the other hand, participants said that household wealth or income is more important for EMIS.

The compensatory potential of the school and its staff is accepted to be exceptionally modest or indeed missing. However, the inquiry about appears that a more comprehensive approach centering on the consistency between the domestic and school environment can make a distinction and the talks made clear that instructors feel like being only a pawn in an instructive framework that’s emphatically influenced by sociodemographic changes within the broader society. On the one hand, instructors ended up demotivated or experience sentiments of futility, whereas on the other hand, teachers’ thoughts approximately the low teachability of ethnic minority understudies are reflected in pupil’s sentiments of futility, demotivation, and indeed mental withdrawal from instruction.

“I feel that high SES is more in the female students, and teachers are not doing discrimination based on a gender level” (EMISS-15).

Vassilakopoulou and Hustad (2021) described that technology-enabled information lacks in terms of SEI. There is digital divide inequality among different socioeconomic representations. The current study found that access to IS s is different among rural schools. Similarly, the study found that removing SEI based on an information technology system should also be accessible to students in rural areas. Additionally, exploitation in the school influences EMIS. Dominant household income students represent their selves in the school. The distribution of economic values showed a significant disparity related to EMIS. These factors mentioned above create dominancy among students, and low SES students were exploited for EMIS home-based and online classes. The theory of neo-Marxism suggests that domination controls the lives and activities of others (Muntaner et al., 2002; Muntaner and Lynch, 2020). In this regard, Wright (2015) described that gaining economic benefits from others is a form of exploitation in society. Our results conclude that SES brings exploitation and domination among students. For instance, the dominant attitude of the students is due to high SES, and these students have access to the EMIS.

## Conclusion

The study aimed to explore the SEI regarding EMIS in rural area students at the middle school level. Our research explores that the SEI present regarding EMIS and household wealth and income brings unequal educational learning among different schools in China.

Moreover, family wealth and SES also affected students’ educational learning in school at home. Family wealth and SES-based exploitation are present in the EMIS among male and female students. Household wealth is significant for the EMIS, and it is recommended to future researchers that a quantitative study should be conducted to measure the exact facts and figures of the amount for the EMIS. The statistical outcomes of SEI research may predict a spatial solution to overcome this problem considerably. However, only a few primary research have been undertaken on the SEI and EMIS in China, and this research is very limited to the schools of Hainan districts as well as not generalizable to the whole Chinese schools. Legal, policy, and information technology-based measures should be arranged for the male and female students as well as data quality and availability for low SES students to overcome the exploitation among rural schools’ students.

Significant in this handle will be bringing the differences displayed in society into the classroom by utilizing social diversification of the staff and the substance of the educational program and to lock in and raise the accountability of all people, communities, and instructive organizations included. Research can offer vital experiences for all performing artists included.

## Practical recommendations

1. Information technology–based data quality and availability for low SES students are mandatory at the middle school level.
2. It is recommended that exploitation could be overcome among rural students if the government provides equal opportunities for access to the EMIS.
3. This study does not generalize to the whole population of China because it is limited to a few schools, not all schools in China.
4. The correlational analysis could be conducted between SEI and EMIS direction for rural Chinese schools.

## Data availability statement

The original contributions presented in this study are included in the article/supplementary material, further inquiries can be directed to the corresponding author.

## Ethics statement

Ethical review and approval was not required for the study on human participants in accordance with the local legislation and institutional requirements. Written informed consent for participation was not required for this study in accordance with the national legislation and the institutional requirements.

## Author contributions

The author confirms being the sole contributor of this work and has approved it for publication.
